# Screening dietary biochanin A, daidzein, equol and genistein for their potential to increase DHA biosynthesis in rainbow trout (*Oncorhynchus mykiss*)

**DOI:** 10.1371/journal.pone.0210197

**Published:** 2019-01-15

**Authors:** Anna Fickler, Stefanie Staats, Gerald Rimbach, Carsten Schulz

**Affiliations:** 1 GMA—Gesellschaft für Marine Aquakultur mbH, Büsum, Germany; 2 Institute of Animal Breeding and Husbandry, Kiel University, Kiel, Germany; 3 Institute of Human Nutrition and Food Science, Kiel University, Kiel, Germany; University of Illinois, UNITED STATES

## Abstract

Plant oil utilization in aquafeeds is still the most practical option, although it decreases the content of the nutritionally highly valuable omega-3 fatty acids eicosapentaenoic acid (20:5n-3, EPA) and docosahexaenoic acid (22:6n-3, DHA) in fish. Phytoestrogens and their metabolites are putatively able to affect genes encoding proteins centrally involved in the biosynthesis of EPA and DHA due to their estrogenic potential. Thus, the aim of the study was to screen the potential of the phytoestrogens to stimulate the biosynthesis of EPA and DHA in rainbow trout (*Oncorhynchus mykiss*). Additionally, the potential effects on growth performance, nutrient composition and hepatic lipid metabolism in rainbow trout were investigated. For that, a vegetable oil based diet served as a control diet (C) and was supplemented with 15 g/kg dry matter of biochanin A (BA), daidzein (DA), genistein (G) and equol (EQ), respectively. These five diets were fed to rainbow trout (initial body weight 83.3 ± 0.4 g) for 52 days. Growth performance and nutrient composition of whole body homogenates were not affected by the dietary treatments. Furthermore, feeding EQ to rainbow trout significantly increased DHA levels by +8% in whole body homogenates compared to samples of fish fed the diet C. A tendency towards increased DHA levels in whole body homogenates was found for fish fed the diet G. Fish fed diets BA and DA lacked these effects. Moreover, EQ and G fed fish showed significantly decreased hepatic mRNA steady state levels for fatty acyl desaturase 2a (delta-6) (*fads2a(d6)*). In contrast, carnitine palmitoyl transferases 1 (*cpt1*) hepatic mRNA steady state levels and hepatic Fads2a(d6) protein contents were not affected by the dietary treatment. In conclusion, when combined with dietary vegetable oils, equol and genistein seem to stimulate the biosynthesis of DHA and thereby increase tissue DHA levels in rainbow trout, however, only to a moderate extent.

## Introduction

The amount of fish oil in feed formulas decreased steadily over the last decades due to increasing prices, resulting in the increased inclusion of plant oils [1,2]. Despite this fact, the share of aquaculture on global fish oil utilization increased, with most of it used for salmonid compound feeds [3]. On the one hand, this is due to the expanded production of these species in aquaculture [4]. On the other hand, producing alternatives high in eicosapentaenoic acid (20:5n-3, EPA) and docosahexaenoic acid (22:6n-3, DHA), such as single cell oils, is still not feasible on a commercial scale due to unreasonably high costs [5,6]. Furthermore, the industry is driven by consumer expectations of farmed fish being a rich source of EPA and DHA [3,5].

In contrast to many other species, salmonids could be reared successfully on diets with up to 100% fish oil replacement [7–11]. Particularly, rainbow trout (*Oncorhynchus mykiss*) can cope with plant oil based diets due to their ability to endogenously biosynthesize omega-3 (n-3) long-chain polyunsaturated fatty acids (LC-PUFA) [12]. Thereby, dietary α-linolenic acid (18:3n-3, ALA), which is found in plant oils like linseed and rapeseed oil [13], is converted to the fatty acids EPA and DHA [14]. This, however, is insufficient to maintain the n-3 LC-PUFA levels of fish fed with fish oil [7,15,16]. Nonetheless, the use of plant oils as alternatives is the most practical and implemented option [17].

Dietary application of phytochemicals seems to be a promising option to counteract the negative effect of plant oil inclusion on the n-3 LC-PUFA content in rainbow trout. Phytochemicals include isoflavones, for example biochanin A, genistein and daidzein [18]. Biochanin A is one of the major isoflavones in red clover (*Trifolium pratense*) [19] whereas genistein and daidzein are naturally present in soy (*Glycine max*) [18]. In contrast, equol is not plant-derived but is a metabolite from daidzein [20]. Equol can be synthesized from daidzein by the intestinal bacterial metabolism in animals and humans [21,22]. Isoflavones and their metabolites can act as antioxidants in humans [23] and showed anti-inflammatory properties in human endothelial cells [24].

Biochanin A, genistein, daidzein and equol belong to the group of phytoestrogens and their metabolites, respectively [20]. The estrogenic potency seems to be attributed to their structural similarity to estradiol [25]. This is of particular interest, since genes encoding proteins involved in the n-3 LC-PUFA biosynthesis were found to be responsive to estrogen [26–28]. For example, hepatic delta-6 desaturase expression was upregulated in response to estradiol in rats [26,29]. The delta-6 desaturase is necessary *inter alia* for synthesizing DHA out of EPA [30]. Kitson et al. [26] suggest that the upregulation of the hepatic delta-6 desaturase expression increased DHA levels in rats. In addition, isoflavones can be potent ligands for the peroxisome proliferator-activated receptor α (PPARα) [31,32]. Carnitine palmitoyl transferase (CPT) 1 is one of the target genes of PPARα and is centrally involved in the biosynthesis of fatty acids [33].

The objective of this study was to screen the potential of dietary isoflavones (Biochanin A, daidzein, genistein) and their metabolites (equol) to stimulate n-3 LC-PUFA biosynthesis and the potential effects on growth in rainbow trout. For this purpose, a feeding trial was conducted with rainbow trout. A vegetable oil based diet was supplemented with the different isoflavones and equol, respectively. Influences of the different dietary treatments on growth performance, fatty acid composition of liver, whole body and fillet, nutrient composition of whole body as well as hepatic fatty acyl desaturase 2a (delta-6) (*fads2a(d6)*) and *cpt1* mRNA steady state levels and hepatic Fads2a(d6) protein levels were investigated.

## Materials and methods

### Experimental setup

The feeding trial was performed at the facilities of the Gesellschaft für Marine Aquakultur mbH (GMA, Büsum, Germany). A total of 300 monosex female rainbow trout juveniles (Forellenzucht Trostadt GbR, Reurieth, Germany) were acclimated to the experimental conditions in a recirculation aquaculture system (RAS, 20 m^3^, turnover rate 2.4 h^-1^, technical oxygen supply). The water purification system consisted of drum filter, biofilter, ultraviolet disinfection and protein skimmer with ozone. A light/dark (12 h/12 h) cycle was adapted. Following a 3-week adaptation period feeding a commercial diet, fish (83.3 ± 0.4 g) were randomly and equally stocked among 15 tanks (volume 150 L, 20 fish per tank, total initial biomass 1.67 ± 0.01 kg). Dietary treatments were randomly distributed in triplicate and were hand fed once per day for 52 days. To adjust daily feed supply of 2.1% of biomass, all tanks were bulk weighed every 14 days. Temperature (16.0 ± 0.6°C), oxygen (10.4 ± 0.2 mg L^-1^ O_2_) and pH (7.4) were monitored continuously. NH_4_^+^ (0.5 ± 0.4 mg L^-1^), NO_2_^-^ (0.5 ± 0.2 mg L^-1^) (Microquant test kit for NH_4_^+^ and NO_2_^-^ Merck KGaA, Darmstadt, Germany) and salinity (6.2 ± 0.2 ‰) were measured daily. The experiment was conducted according to the national regulations for animal welfare (TierSchVersV) and the EU Directive 2010/63/EU for animal experiments. Furthermore, it was approved by the Ministry of Energy, Agriculture, the Environment, Nature and Digitalization (MELUND, Kiel, Germany; approved on 11 July 2017, project number V241-37421/2017).

### Experimental diets

All diets were formulated based on the same feed ingredient composition, only differing in the supplementation of the bioactive substances. A blend of palm fat, rapeseed, linseed and sunflower oil was used as dietary oil source (Table 1). These oil sources were included into the diets to meet the dietary ALA requirement (0.7–1.0% of dry diet) of rainbow trout [13]. Furthermore, only 15% of lean fish meal was used to obtain dietary EPA and DHA levels below the recommended levels for these fatty acids (0.4–0.5% of dry diet) for rainbow trout [13]. Each of the dietary treatments was supplemented with either 1.5 g/kg of dry matter biochanin A (BA), daidzein (DA), equol (EQ) or genistein (G), respectively, in exchange for bentonite. A diet without the addition of bioactive compounds was designed as control diet (C), resulting in five experimental diets in total (C, BA, DA, EQ and G). All diets were isoenergetic and isonitrogenous. Dietary fatty acid composition is shown in Table 2. Amino acid content (not shown) of the diets was calculated based on the amino acid contents of the single ingredients. The experimental diets were designed following the amino acid requirements of rainbow trout [13]. Diets were produced with a pelleting machine (Type 14U175, Amandus Kahl, Hamburg) with 6 mm length and 4 mm in diameter.

**Table 1:** Ingredients and nutrient composition (in g/kg dry matter (DM)) of the experimental diets C, BA, DA, EQ and G.

Diet	C	BA	DA	EQ	G
*Ingredients [g/kg DM]*					
Fish meal ^a^	150	150	150	150	150
Blood meal ^b^	50	50	50	50	50
Feather meal ^c^	55	55	55	55	55
Pea protein isolate ^d^	150	150	150	150	150
Soy protein concentrate ^e^	50	50	50	50	50
Wheat gluten ^f^	140	140	140	140	140
Gelatin ^g^	15	15	15	15	15
Wheat starch ^f^	210	210	210	210	210
***Oil sources***	**115**	**115**	**115**	**115**	**115**
Rapeseed oil ^h^	36	36	36	36	36
Linseed oil ^i^	34	34	34	34	34
Palm fat ^j^	29	29	29	29	29
Sunflower oil ^h^	16	16	16	16	16
Vitamin Mineral premix ^k^	10	10	10	10	10
Calcium hydrogen phosphate ^l^	5.0	5.0	5.0	5.0	5.0
α-Cellulose ^m^	15	15	15	15	15
Lysine ^n^	4.0	4.0	4.0	4.0	4.0
Methionine ^o^	1.0	1.0	1.0	1.0	1.0
Bentonite ^p^	30	28.5	28.5	28.5	28.5
Biochanin A ^q^	-	1.5	-	-	-
Daidzein ^q^	-	-	1.5	-	-
Equol ^q^	-	-	-	1.5	-
Genistein ^q^	-	-	-	-	1.5
*Nutrient composition [g kg*^*-1*^ *DM]*					
Dry matter	891	882	888	893	890
Crude protein	532	535	527	526	535
Crude lipid	166	168	166	166	167
Crude ash	60	58	58	58	59
Total CHO ^r^	242	239	249	250	240
Gross energy [MJ kg^-1^ DM]	23.4	23.4	23.3	23.3	23.3

^a^ Lean fish meal “low ash”, Bioceval GmbH & Co. KG, Cuxhaven, Germany

^b^ Daka porcine bloodmeal, Daka Denmark A/S, Løsning, Denmark

^c^ GePro Goldmehl, GePro Geflügel-Protein Vertriebsgesellschaft mbH & Co. KG, Diepholz, Germany

^d^ Emsland-Stärke GmbH, Emlichheim, Germany

^e^ Euroduna Food Ingredients GmbH, Barmstedt, Germany

^f^ KRÖNER STÄRKE GmbH, Ibbenbüren, Germany

^g^ Gustav Ehlert GmbH & Co. KG, Verl, Germany

^h^ Food store, Büsum, Germany

^i^ Makana Produktion und Vertrieb GmbH, Offenbach a.d. Queich, Germany

^j^ DF 100 PT-PV; EFG Elbe Fetthandel GmbH, Geesthacht, Germany

^k^ Emsland-Aller Aqua GmbH, Golßen, Germany

^l^ JRS Pharma GmbH & Co. KG, Rosenberg, Germany

^m^ Mikro-Technik GmbH & Co. KG, Bürgstadt am Main, Germany

^n^ Biolys, Evonik Industries AG, Essen, Germany

^o^ MetAmino, Evonik Industries AG, Essen, Germany

^p^ Castiglioni Pes y Cía, Buenos Aires, Argentina

^q^ Biochanin A: Cas No: 491-80-5, Daidzein: Cas No: 486-66-8, Equol: Cas No: 531-95-3, Genistein: Cas No: 446-72-0, Xi’an Natural Field Bio-Technique Co., LTD, Xi’an Shaanxi, China

^r^ Total CHO = 1000 –(crude protein + crude lipid + crude ash).

**Table 2:** Fatty acid composition (in % of fatty acid methyl ester (FAME)) of the experimental diets C, BA, DA, EQ and G.

[% of FAME]	C	BA	DA	EQ	G
C14:0	0.7	0.7	0.7	0.7	0.7
C16:0	21.3	21.2	21.3	21.2	21.3
C18:0	3.6	3.6	3.6	3.6	3.6
**Total SFA** ^1^	**26.6**	**26.4**	**26.5**	**26.4**	**26.5**
C16:1	0.5	0.6	0.5	0.6	0.6
C18:1	28.3	28.4	28.4	28.4	28.3
**Total MUFA** ^2^	**32.6**	**32.6**	**32.6**	**32.7**	**32.6**
*n-6*				
C18:2n-6	22.4	22.5	22.5	22.5	22.5
C18:3n-6	0	0	0	0	0
C20:4n-6	0.1	0	0	0	0
*n-3*				
C18:3n-3	15.0	15.2	15.1	15.2	15.1
C18:4n-3	0.2	0.2	0.2	0.2	0.2
C20:5n-3	0.8	0.8	0.8	0.8	0.8
C22:6n-3	1.3	1.3	1.3	1.3	1.3
**Total PUFA** ^3^	**40.4**	**40.6**	**40.4**	**40.6**	**40.4**
ALA [% DM] ^4^	2.3	2.4	2.3	2.3	2.3
∑EPA+DHA [% DM] ^4^	0.3	0.3	0.3	0.3	0.3

^1^ Total SFA is the sum of saturated fatty acids

^2^ Total MUFA is the sum of monounsaturated fatty acids

^3^ Total PUFA is the sum of polyunsaturated fatty acids

^4^ Calculated from the percentage data and the lipid content in the diets (in % DM), assuming 93% of total lipid to be fatty acids.

### Sampling

Eleven acclimatized residual fish (three whole body, eight fillet and liver) were anesthetized, killed by a blow on the head and sampled for determining the initial status. Fish were starved for 72 hours for gastric emptying at the beginning and the end of the 52-day-feeding trial and bulk weighed for determination of growth performance parameters: Feed conversion ratio (FCR): feed intake [g]/weight gain [g]; specific growth rate (SGR, [% d^-1^]): [ln (final body weight)–ln (initial body weight)]/feeding day x 100; protein efficiency ratio (PER): weight gain [g]/protein intake [g]; protein retention efficiency (PRE): 100 x {[(final body protein x final body weight)–(initial body protein x initial body weight)]/protein intake}.

After the bulk weighing, final samples were taken. Both samplings (initial and final) were conducted following the same procedure. Three fish per tank were sacrificed as a pool sample for fatty acid and nutrient composition of whole body homogenate and stored at -20°C. Five fish per tank were used for sampling fillet, liver and spleen. Liver and spleen weights were measured to calculate hepatosomatic and spleen somatic index (Hepatosomatic index (HSI): 100 x (liver weight [g]/body weight [g]); Spleen somatic index (SSI): 100 x (spleen weight [g]/body weight [g]). One part of the liver was taken for measurement of Fads2a(d6) protein levels via ELISA and stored at -80°C. Another part of the liver was used for mRNA quantification via quantitative real-time reverse transcription polymerase chain reaction (qRT-PCR), preserved in RNA later to prevent RNA degradation and stored at -20°C. Residual liver and fillet tissue was pooled for fatty acid analysis and stored at -80°C and -20°C, respectively. Length and weight of all sampled fish was measured to calculate Fulton condition factor (K: 100 x (final body weight x final body length^-3^)). Whole body homogenate and fillet samples were freeze dried (alpha 1–4 LSC; Martin Christ Gefriertrocknungsanlagen GmbH, Osterode am Harz, Germany) and homogenized with a grinding mill (Grindomix, Retsch, Haan, Germany).

### Nutrient composition

Proximate nutrient analysis of ingredients, diets and whole body homogenate was performed at the laboratory of the Gesellschaft für Marine Aquakultur mbH according to EU guideline (EC) 152/2009 [34]. Dry matter was determined by drying samples (ED53 9010–0078; Binder GmbH, Tuttlingen, Germany) until constant mass. Same samples were incinerated in a muffle furnace (LE 6/11/P300; Nabertherm, Lilienthal, Germany) for crude ash analysis. Gross energy content was determined by bomb calorimetry (C 200; IKA-Werke GmbH & Co. KG, Staufen, Germany). Crude lipid content was analyzed according to the Soxhlet method (HYDROTHERM HT 6 and SOXTHERM 416; C. Gerhardt GmbH & Co. KG, Königswinter, Germany). Crude protein content was determined according to the methods of Kjeldahl (Scrubber K-415 and KjelFlex 360; BÜCHI Labortechnik GmbH, Essen, Germany and 877 Titrino plus; Deutsche METROHM GmbH & Co. KG, Filderstadt, Germany).

### Fatty acid composition

Fatty acid composition of diets, liver, fillet and whole body samples was analyzed by LUFA-ITL GmbH, Kiel, Germany via gas chromatography (DGF, C-VI 10 a). Saponification with methanolic NaOH and transmethylation of total lipids was used to prepare fatty acid methyl esters (FAME). For this, boron trifluoride and methanol were utilized (DGF, C-VI 11 a). GC via split-injection (column: CP-Sil 88 50 m x 0.25 mm x 0.2 μm or similar) separated FAME samples. FAME were detected by flame ionization detector (FID) using helium as a carrier gas and subsequently identified in comparison with certified fatty acid standard mixtures. Fatty acid composition was calculated as percent of FAME relative to total FAME.

### RNA isolation and qRT-PCR

Total mRNA was extracted from liver samples of rainbow trout using the Innuprep RNA Mini Kit (Analytik Jena, Jena, Germany) according to the manufacturer’s instructions. Before the total RNA isolation, the samples were homogenized in a TissueLyser II (Qiagen, Hilden, Germany). NanoDrop measurements (NanoDrop2000c; ThermoScientific, Waltham, MA, USA) were used to determine RNA concentration. Quantification of mRNA steady state levels of genes encoding proteins related to lipid metabolism was measured via qRT-PCR. The SensiFast SYBR No-ROX One-Step Kit (Bioline, London, UK) and a Rotor-Gene 6000 real-time PCR cycler (Corbett/Qiagen) were used for that. Primers and their respective annealing temperatures are shown in Table 3. Relative mRNA concentrations were calculated using a standard curve. The mRNA steady state levels of *fads2a(d6)* and *cpt1a/c* were normalized to the expression level of the housekeeping gene elongation factor 1 α (*ef1α*).

**Table 3:** Primer sequences (forward and reverse) and the respective annealing temperatures for mRNA measurements via qRT-PCR of samples from rainbow trout liver.

Genes	Primer sequences FW	Primer sequences Rv	Annealing temp. (°C)	Reference
*ef1α*^a^	ACAAGCCCCTYCGTCTGCC	GCATCTCCACAGACTTSACCTCAG	61	[35]
*fads2a(d6)* ^b^	GCTGGAGARGATGCCACGGA	TGCCAGCTCTCCAATCAGCA	61	[35]
*cpt1a*^c^	TCGATTTTCAAGGGTCTTCG	CACAACGATCAGCAAACTGG	55	[36]
*cpt1c*^c^	CGCTTCAAGAATGGGGTGAT	CAACCACCTGCTGTTTCTCA	59	[36]

^a^ Elongation factor 1 α

^b^ Fatty acyl desaturase 2a (delta-6)

^c^ Carnitine palmitoyl transferase 1.

### Enzyme-linked immunosorbent assay (ELISA)

Fads2a(d6) protein levels were determined using a Fish Fatty Acid Desaturase 2 ELISA Kit (MBS066226, MyBiosource Inc., San Diego, CA, USA; purchased from Biozol, Eching, Germany) following to the manufacturer’s protocol. Liver samples of rainbow trout were diluted in phosphate buffered saline in a TissueLyser II (Qiagen, Hilden, Germany). After the centrifugation, standards and diluted samples were applied to the Microelisa multiwall plate. Samples were incubated and treated with horseradish peroxidase (HRP) conjugate reagent followed by multiple washings. Color intensity was determined at 450 nm using a Labsystems iEMS MF multiplate reader (MTX Lab Systems, Bradenton, FL, USA purchased from Thermo Fisher Scientific, Darmstadt, Germany). The Fads2a(d6) protein concentration in liver samples was calculated via standard curve. Values were normalized to the total protein concentration.

### Statistical analysis

The statistical software R (2017) was used to evaluate the data, including the packages gdata, gplots, lsmeans, multcomp, nlme and piecewiseSEM. Prior to the data evaluation, appropriate statistical models were defined: (1) statistical linear model for data per tank (body weight, DFI, FCR, SGR, PER, PRE, nutrient and fatty acid composition); (2) mixed models [37,38] with tank as random factor if values per fish (HSI, SSI, K, mRNA steady state levels, Fads2a(d6) protein level) were considered. The data were assumed to be normally distributed and to be homoscedastic. These assumptions are based on a graphical residual analysis. The statistical model included the treatment (C, BA, DA, EQ, G). Based on the model, a Pseudo *R*^*2*^ was calculated [39] and multiple contrast tests (many-to-one) (e.g., see [40]) were conducted.

## Results

### Growth, performance and nutrient composition

All groups tripled their initial body weight within the experimental period of 52 days (Table 4). Final body weights did not differ significantly between dietary treatments. In addition, DFI, PER, PRE, SSI and HSI were not significantly affected by the supplementation of bioactive substances. Fish fed with DA had significantly (p = 0.0497) lower K values compared to fish fed the control diet. Furthermore, the nutrient composition analysis of whole body homogenates showed no significant differences between fish fed the diets with bioactive substances and fish fed the control diet (Table 5).

**Table 4:** Growth performance, feed intake, feed efficiency and biometric parameters of rainbow trout fed with the experimental diets C, BA, DA, EQ and G for 52 days.

	C	BA	DA	EQ	G
IBW ^1^	83.4 ± 0.3	83.1 ± 0.3	83.2 ± 0.2	83.2 ± 0.0	83.6 ± 0.5
FBW ^2^	255 ± 1.3	259 ± 3.4	259 ± 6.1	251 ± 6.3	257 ± 4.6
FCR ^3^	0.98 ± 0.01	0.95 ± 0.02	0.95 ± 0.02	0.98 ± 0.02	0.98 ± 0.01
SGR ^4^	2.15 ± 0.01	2.19 ± 0.03	2.19 ± 0.04	2.12 ± 0.05	2.16 ± 0.03
DFI ^5^	2.10 ± 0.02	2.07 ± 0.03	2.08 ± 0.01	2.09 ± 0.01	2.11 ± 0.01
PER ^6^	2.15 ± 0.02	2.24 ± 0.05	2.22 ± 0.05	2.17 ± 0.05	2.15 ± 0.03
PRE ^7^	37.0 ± 0.9	38.0 ± 1.0	38.5 ± 0.4	37.8 ± 0.5	36.9 ± 0.2
SSI ^8^	0.17 ± 0.08	0.15 ± 0.05	0.13 ± 0.05	0.15 ± 0.06	0.16 ± 0.05
HSI ^9^	1.30 ± 0.16	1.29 ± 0.13	1.26 ± 0.14	1.29 ± 0.16	1.21 ± 0.19
K ^10^	1.39 ± 0.08^a^	1.34 ± 0.08	1.32 ± 0.08^b^	1.38 ± 0.10	1.38 ± 0.08

^1^ Average initial body weight [g]

^2^ Average final body weight [g]

^3^ Daily feed intake [% d^-1^]

^4^ Feed conversion ratio = feed intake [g]/weight gain [g]

^5^ Specific growth rate [% d^-1^] = [ln (final body weight)–ln (initial body weight)]/feeding day x 100

^6^ Protein efficiency ratio = weight gain [g]/protein intake [g]

^7^ Protein retention efficiency = 100 x {[(final body protein x final body weight)–(initial body protein x initial body weight)]/protein intake}

^8^ Hepatosomatic index = 100 x (liver weight [g]/body weight [g])

^9^ Spleen somatic index = 100 x (spleen weight [g]/body weight [g])

^10^ Fulton condition factor = 100 x (final body weight x final body length^-3^). Values (mean ± SD, IBW, FBW, FCR, SGR, DFI, PER, PRE: n = 3; HSI, SSI: n = 15 (5 fish/tank); K: n = 24 (8 fish/tank)) with different superscript letters within one row are significantly different (p < 0.05), values without superscript letters within one row are not significantly different (p ≥ 0.05) based on the statistical models described in Materials and Methods.

**Table 5:** Nutrient composition of whole body homogenate (in percent of wet weight (% WW); gross energy in MJ kg^-1^) of rainbow trout before the experiment (Initial) and after being fed the experimental diets C, BA, DA, EQ and G for 52 days.

% WW	Initial	C	BA	DA	EQ	G
Moisture	72.6	67.8 ± 1.2	68.9 ± 0.3	68.3 ± 0.8	68.7 ± 0.3	68.0 ± 0.7
Crude ash	2.7	2.0 ± 0.1	2.2 ± 0.3	2.3 ± 0.1	2.3 ± 0.2	2.2 ± 0.2
Crude protein	16.2	16.9 ± 0.2	16.7 ± 0.1	17.0 ± 0.1	17.0 ± 0.2	16.8 ± 0.1
Crude lipid	8.6	13.3 ± 1.2	12.0 ± 0.2	12.4 ± 0.8	12.0 ± 0.5	12.8 ± 0.8
Gross energy [MJ kg^-1^]	7.2	9.3 ± 0.5	8.8 ± 0.1	9.0 ± 0.3	8.9 ± 0.1	9.2 ± 0.3

Values (mean ± SD, n = 3) without superscript letters within one row are not significantly different (p ≥ 0.05) based on the statistical models described in Methods. Initial data (n = 1, consisting of three fish) was not statistically analyzed.

### Tissue fatty acid composition

Generally, the fatty acid composition of the sampled tissue was not modified to a great extent by the dietary treatments. However, fish fed EQ showed significantly (p = 0.037) increased DHA levels in their whole body homogenate samples compared to the samples of fish fed the diet C. Furthermore, fish fed the diet G tended (p = 0.059) to have higher DHA levels than the fish fed with C (Table 6). In fillet samples, elevated DHA levels were found for EQ and G fed fish (Table 7). In addition, fillets of EQ fed fish showed significantly lower C18:3n-6 and C18:4n-3 levels compared to fish fed the control group (p = 0.017 and p = 0.049, respectively). Levels of SFA, MUFA and PUFA in whole body homogenate and fillet samples were not affected by the dietary treatments and remained in a similar range of concentrations as the initial samples (MUFA > PUFA > SFA). In contrast, livers of fish fed with EQ had significantly higher levels of the fatty acid C18:0 and total SFA (p < 0.0001 and p = 0.021, respectively) (Table 8). Levels of MUFA and PUFA in livers, however, were not affected by the dietary treatments.

**Table 6:** Fatty acid composition (in % of total fatty acid methyl ester (FAME)) of whole body homogenate of rainbow trout before the experiment (Initial) and after being fed the experimental diets C, BA, DA, EQ and G for 52 days.

[% of FAME]	Initial	C	BA	DA	EQ	G
C14:0	1.4	1.2 ± 0.0	1.2 ± 0.0	1.2 ± 0.0	1.2 ± 0.0	1.2 ± 0.0
C16:0	9.3	15.2 ± 0.1	15.2 ± 0.2	15.5 ± 0.5	14.8 ± 0.3	14.8 ± 0.5
C18:0	2.7	3.9 ± 0.1	3.8 ± 0.2	3.9 ± 0.0	3.9 ± 0.1	3.8 ± 0.0
**Total SFA** ^1^	**14.2**	**20.7 ± 0.2**	**20.7 ± 0.3**	**21.1 ± 0.6**	**20.4 ± 0.3**	**20.2 ± 0.6**
C16:1	1.9	4.6 ± 0.3	4.4 ± 0.2	4.5 ± 0.2	4.0 ± 0.2^+^	4.4 ± 0.2
C18:1	43.3	37.3 ± 0.5	37.3 ± 0.1	37.0 ± 0.5	36.8 ± 0.5	37.4 ± 0.7
**Total MUFA** ^2^	**54.0**	**47.9 ± 0.7**	**47.6 ± 0.2**	**47.3 ± 0.5**	**47.1 ± 0.4**	**47.8 ± 0.9**
*n-6*						
C18:2n-6	15.0	15.2 ± 0.4	15.5 ± 0.2	15.4 ± 0.1	15.6 ± 0.3	15.4 ± 0.3
C18:3n-6	0.6	0.5 ± 0.0	0.5 ± 0.0	0.5 ± 0.0	0.5 ± 0.0	0.6 ± 0.0
C20:4n-6	0.7	0.6 ± 0.0	0.6 ± 0.0	0.6 ± 0.0	0.6 ± 0.0	0.6 ± 0.0
*n-3*						
C18:3n-3	3.9	6.7 ± 0.3	6.6 ± 0.2	6.6 ± 0.0	6.7 ± 0.2	6.6 ± 0.3
C18:4n-3	1.1	1.1 ± 0.0	1.1 ± 0.0	1.2 ± 0.0	1.1 ± 0.1	1.2 ± 0.1
C20:5n-3	1.3	0.7 ± 0.0	0.7 ± 0.0	0.7 ± 0.0	0.7 ± 0.0	0.7 ± 0.0
C22:6n-3	6.2	3.9 ± 0.1^a^	3.9 ± 0.1	3.8 ± 0.1	4.2 ± 0.1^b^	4.2 ± 0.1^(b)^
**Total PUFA** ^3^	**31.3**	**31.3 ± 0.7**	**31.4 ± 0.2**	**31.1 ± 0.5**	**31.9 ± 0.4**	**31.7 ± 0.9**

^1^ Total SFA is the sum of saturated fatty acids

^2^ Total MUFA is the sum of monounsaturated fatty acids

^3^ Total PUFA is the sum of polyunsaturated fatty acids. Values (mean ± SD, n = 3, consisting of three fish each) with different superscript letters within one row are significantly different (p < 0.05), values without superscript letters within one row are not significantly different (p ≥ 0.05) based on the statistical models described in Materials and Methods.

^(b)^ Brackets indicate a tendency towards a statistical difference (p < 0.1). Initial data (n = 1, consisting of three fish) was not statistically analyzed.

**Table 7:** Fatty acid composition (in % of total fatty acid methyl ester (FAME)) of fillet of rainbow trout before the experiment (Initial) and after being fed the experimental diets C, BA, DA, EQ and G for 52 days.

[% of FAME]	Initial	C	BA	DA	EQ	G
C14:0	1.2	1.2 ± 0.0	1.2 ± 0.1	1.1 ± 0.0	1.2 ± 0.0	1.2 ± 0.0
C16:0	9.8	15.8 ± 0.2	15.6 ± 0.4	15.5 ± 0.0	15.8 ± 0.4	15.6 ± 0.0
C18:0	3.0	3.8 ± 0.1	3.7 ± 0.1	3.8 ± 0.0	3.9 ± 0.1	3.9 ± 0.0
**Total SFA** ^1^	**14.7**	**21.3 ± 0.1**	**21.1 ± 0.4**	**21.1 ± 0.3**	**21.4 ± 0.4**	**21.1 ± 0.1**
C16:1	1.5	4.4 ± 0.1	4.4 ± 0.3	4.3 ± 0.3	4.5 ± 0.1	4.1 ± 0.0
C18:1	41.1	35.1 ± 0.3	35.1 ± 0.3	35.1 ± 0.4	34.4 ± 0.2^+^	34.7 ± 0.1
**Total MUFA** ^2^	**50.7**	**45.0 ± 0.2**	**45.2 ± 0.0**	**45.0 ± 0.6**	**44.7 ± 0.3**	**44.3 ± 0.0**
*n-6*						
C18:2n-6	14.2	15.0 ± 0.2	15.1 ± 0.1	15.1 ± 0.0	15.0 ± 0.2	15.3 ± 0.1
C18:3n-6	0.5	0.6 ± 0.0^a^	0.5 ± 0.0	0.5 ± 0.0	0.4 ± 0.0^b^	0.6 ± 0.1
C20:4n-6	0.2	0.7 ± 0.0	0.7 ± 0.0	0.7 ± 0.0	0.7 ± 0.0	0.7 ± 0.0
*n-3*						
C18:3n-3	3.4	6.5 ± 0.1	6.6 ± 0.1	6.6 ± 0.0	6.7 ± 0.0	6.6 ± 0.2
C18:4n-3	0.9	1.4 ± 0.0^a^	1.2 ± 0.0	1.2 ± 0.0	1.1 ± 0.1^b^	1.3 ± 0.2
C20:5n-3	1.6	0.9 ± 0.0	0.9 ± 0.0	0.9 ± 0.0	0.9 ± 0.0	0.9 ± 0.0
C22:6n-3	10.2	5.7 ± 0.1	5.6 ± 0.1	5.8 ± 0.2	5.9 ± 0.2	6.1 ± 0.2
**Total PUFA** ^3^	**34.4**	**33.4 ± 0.1**	**33.4 ± 0.3**	**33.4 ± 0.3**	**33.4 ± 0.5**	**34.1 ± 0.1**

^1^ Total SFA is the sum of saturated fatty acids

^2^ Total MUFA is the sum of monounsaturated fatty acids

^3^ Total PUFA is the sum of polyunsaturated fatty acids. Values (mean ± SD, n = 3, consisting of fillets from five fish each) with different superscript letters within one row are significantly different (p < 0.05), values without superscript letters within one row are not significantly different (p ≥ 0.05) based on the statistical models described in Materials and Methods. Initial data (n = 1, consisting of fillets from eight fish) was not statistically analyzed.

**Table 8:** Fatty acid composition (in % of total fatty acid methyl ester (FAME)) of liver of rainbow trout before the experiment (Initial) and after being fed the experimental diets C, BA, DA, EQ and G for 52 days.

[% of FAME]	Initial	C	BA	DA	EQ	G
C14:0	0.7	0.8 ± 0.1	0.9 ± 0.1	0.8 ± 0.0	0.7 ± 0.0	0.8 ± 0.1
C16:0	15.9	14.6 ± 0.8	14.8 ± 0.1	14.4 ± 0.3	14.7 ± 0.3	14.7 ± 0.8
C18:0	5.4	8.1 ± 0.2^a^	7.8 ± 0.1	7.7 ± 0.4	10.2 ± 0.0^b^	7.7 ± 0.7
**Total SFA** ^1^	**23.3**	**24.2 ± 1.0**^**a**^	**24.3 ± 0.1**	**23.4 ± 0.3**	**26.3 ± 0.2**^**b**^	**23.8 ± 1.0**
C16:1	0.8	2.7 ± 0.1	3.4 ± 0.3	3.1 ± 0.4	2.1 ± 0.1	3.1 ± 1.0
C18:1	20.7	21.7 ± 2.6	23.0 ± 1.0	23.2 ± 2.6	16.3 ± 0.6	23.2 ± 6.2
**Total MUFA** ^2^	**25.5**	**30.2 ± 2.7**	**32.7 ± 1.4**	**32.3 ± 3.1**	**24.7 ± 0.4**	**31.8 ± 8.0**
*n-6*						
C18:2n-6	7.2	5.6 ± 0.5	5.3 ± 0.0	5.8 ± 0.4	5.5 ± 0.6	6.1 ± 0.2
C18:3n-6	0.2	0.2 ± 0.0^a^	0.2 ± 0.0	0.2 ± 0.0	0.2 ± 0.0	0.2 ± 0.0^b^
C20:4n-6	4.7	5.8 ± 0.4^a^	5.4 ± 0.1	5.7 ± 0.3	7.3 ± 0.4	3.5 ± 2.3^b^
*n-3*						
C18:3n-3	1.7	1.4 ± 0.2	1.1 ± 0.0	1.3 ± 0.2	1.5 ± 0.2	1.5 ± 0.1
C18:4n-3	0.5	0.3 ± 0.1	0.3 ± 0.0	0.3 ± 0.0	0.3 ± 0.0	0.3 ± 0.0
C20:5n-3	3.9	2.2 ± 0.1	2.1 ± 0.0	1.9 ± 0.2	2.3 ± 0.3	2.2 ± 0.4
C22:6n-3	29.5	25.2 ± 2.0	23.2 ± 1.2	23.7 ± 1.8	26.5 ± 0.9	23.8 ± 4.3
**Total PUFA** ^3^	**50.8**	**45.4 ± 1.7**	**42.7 ± 1.3**	**43.8 ± 2.8**	**48.7 ± 0.6**	**44.1 ± 6.8**

^1^ Total SFA is the sum of saturated fatty acids

^2^ Total MUFA is the sum of monounsaturated fatty acids

^3^ Total PUFA is the sum of polyunsaturated fatty acids. Values (mean ± SD, n = 3, consisting of livers from five fish each) with different superscript letters within one row are significantly different (p < 0.05), values without superscript letters within one row are not significantly different (p ≥ 0.05) based on the statistical models described in Materials and Methods. Initial data (n = 1, consisting of eight livers) was not statistically analyzed.

### Hepatic *fads2a(d6)* mRNA steady state and fads2a(d6) protein levels

**Fig 1:**
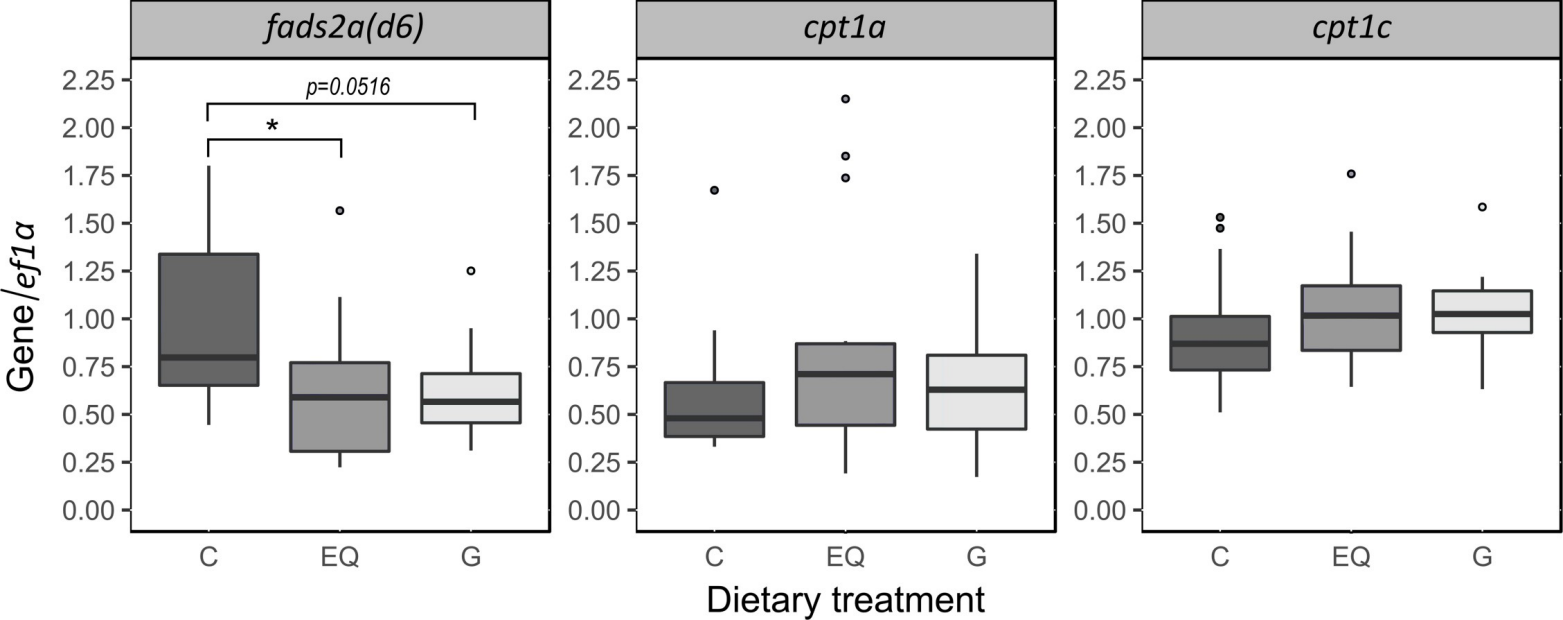
Hepatic mRNA steady state levels. Rainbow trout were fed the experimental diets C, EQ and G for 52 days. Presented are boxplots of relative liver mRNA levels of fatty acyl desaturase 2a (delta-6) (*fads2a(d6))* and carnitine palmitoyl transferase 1 (*cpt1*) a and c, respectively. Hepatic mRNA steady state levels were determined by qRT-PCR analysis and normalized to the housekeeping gene *ef1α*. Boxes represent values between the 25^th^ and the 75^th^ percentile; whiskers indicate 1.5 SD; medians are indicated by solid lines; outliers (above/below 1.5 SD) are indicated by solid circles. At the end of the experiment, 15 individuals per treatment were sampled in total (n = 15). Statistically significant differences between dietary treatments are represented by asterisks; p < 0.05 (*) based on the statistical models described in Materials and Methods.

**Fig 2:**
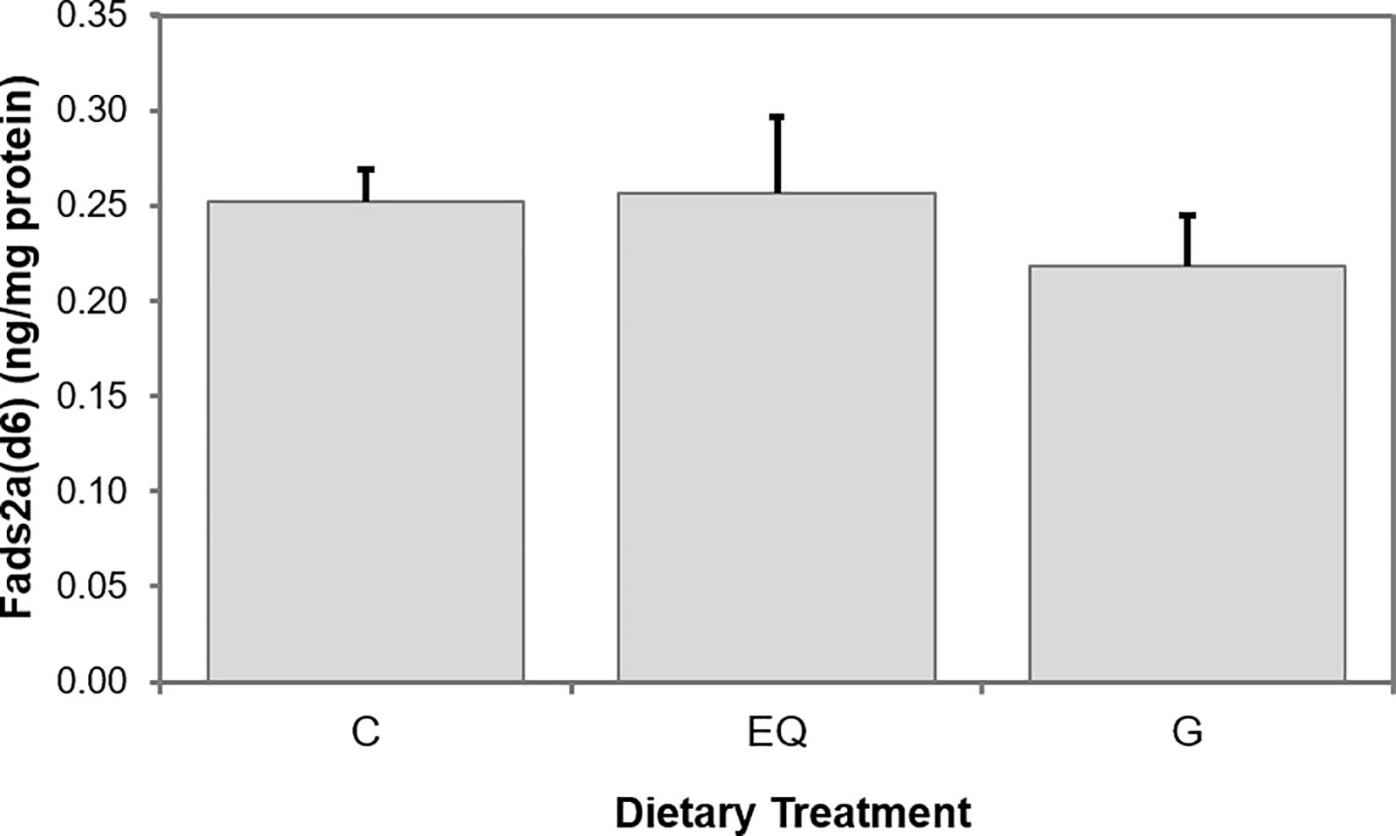
Fatty acyl desaturase 2a (delta-6) (Fads2a(d6)) protein levels in liver samples. Rainbow trout were fed the experimental diets C, EQ and G for 52 days. **Fads2a(d6)** protein levels were determined by ELISA and were normalized to the total protein level (ng/mg protein). Bars represent the final expression values. At the end of the experiment, four individuals per treatment in total were sampled randomly (n = 4). Values were tested on significance based on the statistical models described in Material and Methods. No significant differences were found.

Fish fed the diet EQ showed significantly (p = 0.049) decreased and fish fed the diet G tended (p = 0.052) to have lower hepatic *fads2a(d6)* mRNA steady state levels compared to fish fed the control group. *Cpt1a* and *cpt1c* levels were similar between the dietary treatments (Fig 1). Furthermore, hepatic Fads2a(d6) protein levels of rainbow trout were not affected by the dietary treatment (Fig 2).

## Discussion

In the present study, fish responded well to their dietary treatments and tripled their initial body weight within 52 days of feeding. The FCR (< 1.0) and SGR values (> 2.1%/d) can be considered as reasonable since other studies reported similar, lower (SGR) and higher (FCR) values for rainbow trout of the same size [16,41,42]. No significant effect on growth performance was found between fish of any of the treatments in the present study. For EQ and G fed fish, these findings are in line with results in literature. Jourdehi et al. [43] also found no effect on growth in female beluga sturgeon fed with equol. Furthermore, Torno et al. [42] reported no negative impact on growth performance of rainbow trout fed genistein. In contrast, Crespillo et al. [44] found a reduction of body weights in rats fed a diet containing daidzein. A possible explanation for the lack of effect of the bioactive substances could be their dietary dosage utilized in the present study. Phytoestrogens have been shown to affect components of the growth hormone (GH)/insulin-like growth factor (IGF) axis in livers of rainbow trout [45]. The negative impact of genistein on the GH/IGF axis was enhanced when genistein was administered at a higher concentration [45]. The dose of 1.5 g/kg DM dietary genistein seemed to have no effect on the growth performance of rainbow trout in the present study and could thus be considered as a low dietary concentration. With regard to the observed effects in the present study, this assumption might also be true for equol, daidzein and biochanin A. Moreover, the feed conversion ratios were not affected in response to dietary bioactive compounds. Similar results were reported by Torno et al. [42] who fed genistein at 3.0 g/kg DM to rainbow trout. According to this study, dietary genistein did not affect feed conversion negatively but induced a significant reduction in feed intake. The authors assume that the bitterness of phytochemicals partly contributed to the decrease in feed intake. In the present study, dietary bioactive substances did not alter feed intake when daily feeding levels were set at 2.1% of biomass per day. A direct comparison is difficult, because fish in the present study were fed on a fixed daily feeding level that was below the reported feed intakes in the study of Torno et al. [42]. Furthermore, the dietary genistein concentration of 3.0 g/kg DM was twice the amount of the concentration used in the present study (1.5 g/kg DM). Thus, it seems that supplementation of 1.5 g/kg DM of these bioactive substances does not affect the palatability of the diets and does not influence feed intake negatively. Furthermore, PER and PRE values of fish as well as the nutrient composition of whole body homogenate samples were not affected by dietary bioactive substances in the present study. This is contrasting previous results of Torno et al. [42] who described significantly increased PER values and protein productive values (PPV, equals PRE) for rainbow trout fed with 3.0 g/kg DM of genistein. This, again, strongly supports the assumption that phytoestrogens and their metabolites might exhibit dose-dependent effects. In addition, Schiller Vestergren et al. [46] assumed that differences in the response to bioactive compounds could be due to varying physiological parameters (species, fish size, gender, age) and environmental conditions (temperature, feed composition). The observations in the present study indicate that dietary equol, genistein, daidzein and biochanin A at 1.5 g/kg DM can be fed to rainbow trout without detrimental effects on growth and performance. Furthermore, any interactions due to the inclusion of soy protein concentrate into the diets are expected to be negligible. The overall content of isoflavones in soy is low with most of them being present as glucosinolates [47]. Glycosidic bond isoflavones need to be hydrolized in the intestine before absorption [47], requiring additional time for this process in the intestine [48]. Thus, we assumed that these substances might be mostly excreted and the amount of isoflavones being bioavailable can be considered as low.

The fatty acid composition of whole body homogenates and fillets was not affected to a great extent by the supplementation of bioactive substances in the present study. However, the DHA levels were increased in whole body homogenates and fillet samples of fish fed EQ and G in comparison to samples of fish fed the diet C. In contrast, EPA levels remained unaffected by the dietary treatments. For fish fed EQ, the increase in DHA could be possibly explained by its estrogenic potential [49]. In vertebrates, the synthesis of DHA occurs via the elongation (elongase 2) of C22:5n-3 to C24:5n-3 followed by a desaturation (delta-6 desaturation) to C24:6n-3 and a final peroxisomal β-oxidation [50]. The genes encoding for elongase 2 (*elovl2*) and the delta-6 desaturase (*fads2*) have been reported to be responsive to estrogen [26,28]. Estrogen application increased DHA in rats [26] and humans [51] by increasing the expression of *fads2*. Furthermore, equol showed a greater estrogenic potency than genistein, daidzein and biochanin A (equol > genistein > daidzein > biochanin A) in rainbow trout hepatocyte cultures [49]. In contrast to genistein and daidzein, equol is metabolically inert and can be readily absorbed [21]. This results inter alia in a higher bioavailability of equol in comparison to that of genistein [52]. Thus, the combination of the higher estrogenic potency and the increased bioavailability of equol putatively enhances the effect on the biosynthesis of DHA in comparison to the other bioactive substances in the present study. Simultaneously, the supplied amount of genistein, biochanin A and daidzein might be too low to develop estrogen- like effects. The significantly increased levels of the fatty acid C18:0 in the livers of fish fed EQ obtained in the present study support this hypothesis. For instance, an increase of C18:0 fatty acid has also been associated with increasing estrogen levels in rats [53]. Thus, it seems that dietary equol might have influenced the lipid metabolism via estrogen-like mechanisms. As the increase of both, DHA and C18:0 was only found for fish fed EQ it seems reasonable that other mechanisms led to the increased DHA levels in the G treatment. A factor possibly contributing to an increase in DHA are the antioxidant properties of genistein [23,54]. For example, genistein was found to protect cells from oxidative stress induced damage [55]. In the study of Hernandez-Montes et al. [55], genistein activated the transcription factor Nrf1 and thereby increased the activity and expression of its target gene glutathione peroxidase, an enzyme involved in cellular defense mechanisms in endothelial cells. Thus, in the present study, genistein could have also increased the expression of glutathione peroxidase and thereby protected DHA from oxidation, leading to an indirect increase of DHA concentrations. However, bioactive substances could have further mechanisms of action to affect the DHA levels in fish. Trattner et al. [56] suggested that increased DHA levels in Atlantic salmon hepatocytes treated with the phytochemical sesamin might be a result of an increased peroxisomal β-oxidation. The peroxisomal β-oxidation is regulated inter alia by the transcription factor PPARα: PPARα binds to a peroxisome proliferator response element, thereby regulating the transcription of target genes centrally involved in the peroxisomal β-oxidation [57]. Ligands can bind to and activate PPARα. Genistein and daidzein have been found to be potent ligands for PPARα [31,32]. However, fish fed the diet G only tended to increase DHA levels in whole body homogenates and fish fed DA lacked an effect on tissue DHA in the present study. Thus, one could hypothesize that affecting the biosynthesis of DHA via PPARα is more complex and takes longer than directly influencing it via estrogen-like mechanisms. Furthermore, it could be possible that the dietary dosage of 1.5 g/kg DM of biochanin A and daidzein are too low to develop an effect either way.

The increase in DHA levels in whole body homogenates was not shown to the same extent in fillet samples of fish fed EQ and G diets. This could be partly explained by the very slow synthesis of DHA from ALA in rainbow trout [12]. Thus, it could be possible that the experimental duration in the present study was too short. It remains elusive whether fish fed EQ and G diets could have further increased their DHA fillet levels if the experimental period was longer. However, the effect of dietary equol and genistein on DHA tissue levels was only moderate. Thus, further investigations are needed to evaluate if and to what economically reasonable extent these bioactive substances can be included into rainbow trout diets to result in similar EPA and DHA levels that would be expected from fish fed a diet based on fish oil.

In the present study, hepatic mRNA steady state levels of *fads2a(d6)* were significantly lower in samples of fish fed EQ and tended to be lower in fish fed G compared to fish fed the diet C. Estrogen treatment enhanced the hepatic delta-6 desaturase expression and resulted in increased DHA levels in rats [26]. Thus, DHA concentrations in tissue samples were not well supported by the hepatic mRNA steady state levels. This effect has already been described in Atlantic salmon hepatocytes treated with the phytochemical sesamin [56]. In this study, DHA levels in hepatocytes were also significantly increased, although the gene expression of the gene encoding the delta-6 desaturase was down-regulated. A factor possibly contributing to the results in the present study could be the starvation time of fish before sampling. The half-life of genistein in rainbow trout was reported to be 13 hours [58]. The authors suggest that the half-life of genistein in trout is similar to the values obtained in humans. Assuming that this is also applicable to other phytoestrogens, equol could have a half time of less than ten hours in trout as it was reported for humans [21]. Therefore, it could be possible that by the time of sampling most of the genistein and equol has already been excreted by the fish. In addition, Schiller Vestergren et al. [59] assume that mRNA turnover responds to dietary changes. Thus, mRNA levels might fluctuate constantly. Another factor leading to the decreased hepatic *fads2a(d6)* mRNA steady state levels in the EQ and G fed groups could be the influence of LC-PUFA on gene expression. A negative feedback loop from n-3 LC-PUFA on the delta-6 desaturase mRNA expression has been found in zebrafish [60]. In the present study, DHA levels in whole body homogenate and fillet samples were highest in fish fed diets with EQ and G, respectively. Therefore, one could hypothesize that by the time of sampling, the increased DHA tissue levels in fish fed the EQ and G led to a negative feedback on *fads2a(d6)* mRNA expression. Interestingly, the Fads2a(d6) protein levels did not show significant differences between the dietary treatments. This mismatch of *fads2a(d6)* mRNA steady state and Fads2a(d6) protein levels has also been reported by Torno et al. [61] in a study with rainbow trout fed the phytochemical resveratrol. However, we cannot exclude putative effects of the bioactive substances on the delta-6 desaturase activity, as this factor was not analyzed in the present study. Furthermore, *cpt1a* and *cpt1c* mRNA steady state levels in the present study were not affected by the dietary treatments. Cpt1 is involved in the mitochondrial β-oxidation. The isoforms Cpt1a and Cpt1c are necessary for the uptake of long-chain fatty acids into the mitochondria [33]. Our results are contrasting the findings of Kim et al. [31], who reported that genistein induced the expression of Cpt1 via activating PPARα. Furthermore, these authors suggested that this effect was non-estrogenic and dose-dependent. Again, it seems that the dietary dosage of bioactive substances is important and needs to be considered when applied in rainbow trout.

## Conclusion

The present study indicates that dietary equol, genistein, daidzein and biochanin A can be fed to rainbow trout at 1.5 g/kg DM without detrimental effects on growth and performance parameters. Furthermore, 1.5 g/kg DM of dietary equol increased DHA levels putatively by influencing the biosynthesis via estrogen-like mechanisms. However, this was not resembled in hepatic *fads2a(d6)* mRNA steady state levels and protein contents. In contrast, genistein seems to affect the biosynthesis of DHA either indirectly due to its antioxidative characteristics or via PPARα-mediated pathways. Biochanin A and daidzein did not affect the tissue fatty acid composition of rainbow trout. However, the increase in DHA levels due to dietary equol and genistein were only moderate. The dietary dosage of these substances might be an important factor influencing the extent of the effect on the biosynthesis of DHA. Thus, future studies should investigate dose-dependent effects of these bioactive substances on growth performance and LC-PUFA synthesis in rainbow trout.
